# Age-related waning of immune responses to BCG in healthy children supports the need for a booster dose of BCG in TB endemic countries

**DOI:** 10.1038/s41598-018-33499-4

**Published:** 2018-10-17

**Authors:** Elizabeth Whittaker, Mark P. Nicol, Heather J. Zar, Nontobeko G. Tena-Coki, Beate Kampmann

**Affiliations:** 1 Academic Department of Paediatrics, St Marys Campus, Imperial College London, 2nd Floor Wright-Fleming Building, Norfolk Place, London, W2 1PG UK; 2 Division of Medical Microbiology, Department of Pathology And Institute of Infectious Disease and Molecular Medicine UCT Faculty of Health Sciences, Observatory, 7925 South Africa; 3 MRC Unit of Child & Adolescent Health, University of Cape Town, Rondebosch, Cape Town, 7701, South Africa; 4 Department of Paediatrics & Child Health, Red Cross War Memorial Children’s Hospital, Klipfontein Road, Rondebosch, Cape Town 7701 South Africa; 5 Vaccines & Immunity Theme MRC Unit The Gambia Fajara, Serrekunda, West Africa The Gambia

## Abstract

In the absence of a more effective vaccine against TB and in the interest of developing one, it is essential to understand immune responses associated with BCG protection. We comprehensively characterized T cell populations in BCG-vaccinated children over time. Blood from 78 healthy, BCG-vaccinated children representing four age groups (<1 yr, ≥1 yr <2 yr, ≥2 yr <5 yr, ≥5 yr), was stimulated *in vitro* for 24 hours and 6 days with live BCG to induce effector and central memory responses. Antigen-specific CD4, CD8, γδ and regulatory T cell populations were phenotyped and intracellular and secreted cytokines measured by flow cytometry and multiplex ELISA respectively. Our results demonstrated that populations of naïve T cells predominated in infants, compared to older children. However, BCG-specific effector CD4 T cell responses were equivalent and antigen-specific CD4 T cell proliferative capacity was increased in infants compared to older children. Increases in innate immune responses including γδ T cell responses and secreted pro-inflammatory cytokines were noted with increasing age. In conclusion, we identified that the capacity to expand and differentiate effector T cells in response to BCG stimulation wanes with increasing age, which may indicate waning central memory immunity. Booster vaccination could be considered to maintain the antigen-specific central memory pool and possibly enhance the duration of protection.

## Introduction

A protective vaccine with higher efficacy than BCG remains an essential tool to combat the ongoing tuberculosis (TB) epidemic^1^. In the meantime, BCG remains a widely-used vaccine with varying protection against pulmonary TB (0–80%), but proven efficacy against disseminated disease in children under 5 years of age^2^. Despite its widespread use for nearly a century, the mechanisms of immune protection imparted by BCG remain poorly understood. Understanding how BCG confers protection - or fails to do so- is central to the development of new vaccines that aim to either boost its efficacy or replace it altogether^3^, and correlates of immune protection against TB still remain elusive.

In addition to epidemiological risk factors such as close contact between infants and their TB-transmitting caregivers, increasing immune maturation is likely to underlie the observation that the risk of severe manifestations of TB decrease with increasing age, and is comparable with adults by the age of 5 years^4^. Correlates of protection are therefore likely to be distinct between adults and infants.

The role of IFNγ as an immune correlate of protection induced by BCG vaccination has been questioned, particularly through a large cohort study of BCG vaccinated South African infants^5^. Murine data support the hypothesis that the balance between mycobacterial antigen-specific IL17 and IFNγ producing T cells is of importance in mediating BCG-induced protection^6^ but gaps in knowledge remain in the context of such responses in children. Neonates are recognized to have diminished IFNγ production compared to adults, which may contribute to their susceptibility to disseminated disease, this may be associated with raised levels of Th17-associated cytokines. Furthermore, infants have increased numbers of circulating regulatory T cells which may contribute to lower levels of IFNγ and susceptibility to TB disease^7,8^.

Immune cell populations and effector molecules induced by BCG vaccination have been described in cross-sectional studies from a variety of countries, including in children of different ages^5,9–11^. These include CD8+ T cells, γδ T cells, Th17 cells, polyfunctional T cells and regulatory T cells, but to date no studies of the longevity of the responses have been published.

We therefore characterized age-related antigen-specific and non-specific effector and central memory responses to BCG, including Th1, Th17 and regulatory T cell populations and their associated cytokines in children to examine why BCG vaccine efficacy wanes with age.

## Methods

### Study setting

The study was conducted at the Red Cross War Memorial Children’s Hospital (RCH) in Cape Town, South Africa. Ethical approval was obtained from the Faculty of Health Sciences Research Ethics Committee at the University of Cape Town (HREC: 062/2011). Following written informed consent from the legal guardian, blood samples were taken. The study was carried out in accordance with the local regulations.

### Eligibility

Healthy children who presented to RCH for elective surgical interventions were recruited for this study. A medical and TB contact history was documented prior to recruitment. Only children with a written record of BCG vaccination (BCG Denmark strain) at birth were included. Exclusion criteria were a history of significant household contact with TB; previous treatment for TB; recurrent infections or hospital admissions; persistent cough for longer than 2 weeks; intercurrent febrile illness; failure to thrive or known immunodeficiency including HIV infection. If HIV status was unknown, an HIV test was performed following counselling. All children were screened for *Mycobacterium tuberculosis* sensitisation using Quantiferon TB Gold In Tube (Cellestis, Carnegie, Australia). Only healthy children without evidence of *M. tuberculosis* sensitization were included.

### Blood collection, stimulation and cryopreservation

0.5 ml of heparinised blood was incubated within 4 hours of collection with BCG (SSI strain, 5 × 10^5^ colony forming units (cfu)/ml), as previously described^12^. Medium alone served as negative control; staphylococcal enterotoxin B (SEB 10 µg/ml final concentration; Sigma, UK) was used as a positive control. Brefeldin-A was added for the last 5 hours of the 20-hour incubation. Cells were then harvested, fixed, and cryopreserved.

A further 0.25 ml aliquot of whole blood (diluted 1:10 in RPMI 1640) was incubated with BCG (SSI, 2.5 × 10^6^ cfu/ml) for 6 days for a Ki67 lymphoproliferation assay as previously described^13^ or with medium alone or SEB (5 µg/ml final concentration) as negative and positive controls respectively. To assess intracellular cytokine production, 10 ng/mL phorbol 12-myristate 13-acetate (PMA, Sigma-Aldrich), 1.5 µg/mL ionomycin (Sigma-Aldrich) and 1.5 µg/mL Brefeldin A (Sigma-Aldrich) were added during the last 5 h of culture. Cells were then harvested, stained with a viability dye, fixed and cryopreserved.

Prior to the addition of PMA/Ionomycin/Brefeldin, 500 μl of supernatant was removed and stored at −80 °C for subsequent analysis by multiplex ELISA (Bio-plex Pro^TM^ Human Th17 Cytokine Panel) to determine levels of secreted chemokines and cytokines.

### Cell staining and flow cytometric analysis

Cryopreserved cells were thawed, washed, and permeabilized with Perm/wash solution (BD Biosciences). Cells were then incubated at 4 °C for 1 hour with fluorescence-conjugated antibodies directed against surface antigens and intracellular cytokines. For detection of the transcription factor FOXP3, a nuclear permeabilisation buffer (eBioscience) was used in place of the Perm/Wash solution as per manufacturer’s protocol. The following fluorescence-conjugated antibodies were used: anti-CD3 PacBlue, anti-GDTCR PE, anti-CD27 PECy7, anti-CD25 APC, anti-IFNγ Alexafluor 700, anti-IL17A Alexafluor 647, anti-Ki67FITC (all BD biosciences, San Jose, CA); anti-CD45RA QDot655, anti-CD8 QDot605, anti-CD4 QDot605 (all Invitrogen, Eugene, Or); anti-CD39 FITC, anti-IL22 PerCP-Efluor710, anti-FOXP3 PE (all eBioscience, SanDiego, CA).

The entire sample was acquired on a BD LSR Fortessa Flow Cytometer (model 649225B7, power 690 W, manufactured Sept 2010) configured with 4 lasers and 22 detectors using FACSDiva software (BD Bioscience, San Jose, CA, USA). Compensation for overlap of fluorescence detection was performed using compensation beads. Multiparameter panel development included evaluation of appropriate staining controls of antibody and fluorochrome interactions and of spectral overlap using control blood samples.

### Secreted cytokine measurement

Concentrations of IL1β, IL4, IL6, IL10, IL17, IL22, IL23, IFNγ, TNFα were measured in supernatants from whole blood stimulated for 6 days by Milliplex MAP Multiplex Immunoassay (based on Luminex MAP technology; Millipore) on a Bio-Rad Luminex 100 Bio-Plex Liquid Array Multiplexing System Fluorescent Reader, according to the manufacturer’s instructions. The following concentrations of the standards diluted in 200 μl assay buffer were used: 10000, 2000, 400, 80, 16, and 3.2 pg/ml.

### Data analysis

Multi-parameter flow cytometry data was analysed using FlowJo v 9.4.11 (TreeStar, Ashland, OR). Combinations of cytokine-producing cells were determined by Boolean Gating in FlowJo.

SPSS and GraphPad Prism were used for statistical analysis. Negative control (background) values for cytokine expression were subtracted from BCG-induced responses. An empiric cut-off value of 0.01% was interpreted as a positive response in line with previous publications^14^. Differences between groups were calculated using either Mann-Whitney or Kruskal-Wallis analysis of variance. For correlations, a Spearman coefficient for non-parametric data was calculated. All tests were two-tailed and a value of p < 0.05 was considered significant.

## Results

78 children were recruited to the study, stratified in 4 age groups of comparable sizes and reflective of the reported age-dependent susceptibility to TB disease (group 1: <1 yr n = 19, group 2: ≥1 yr < 2 yr n = 18, group 3: ≥2 yr < 5 yr n = 22, group 4: ≥5 yr n = 19). Malnutrition, as measured by z-score was uncommon in this cohort. All children were fully vaccinated according to the South African schedule.

### Age-dependent SEB and BCG-induced T cell responses

**Figure 1:**
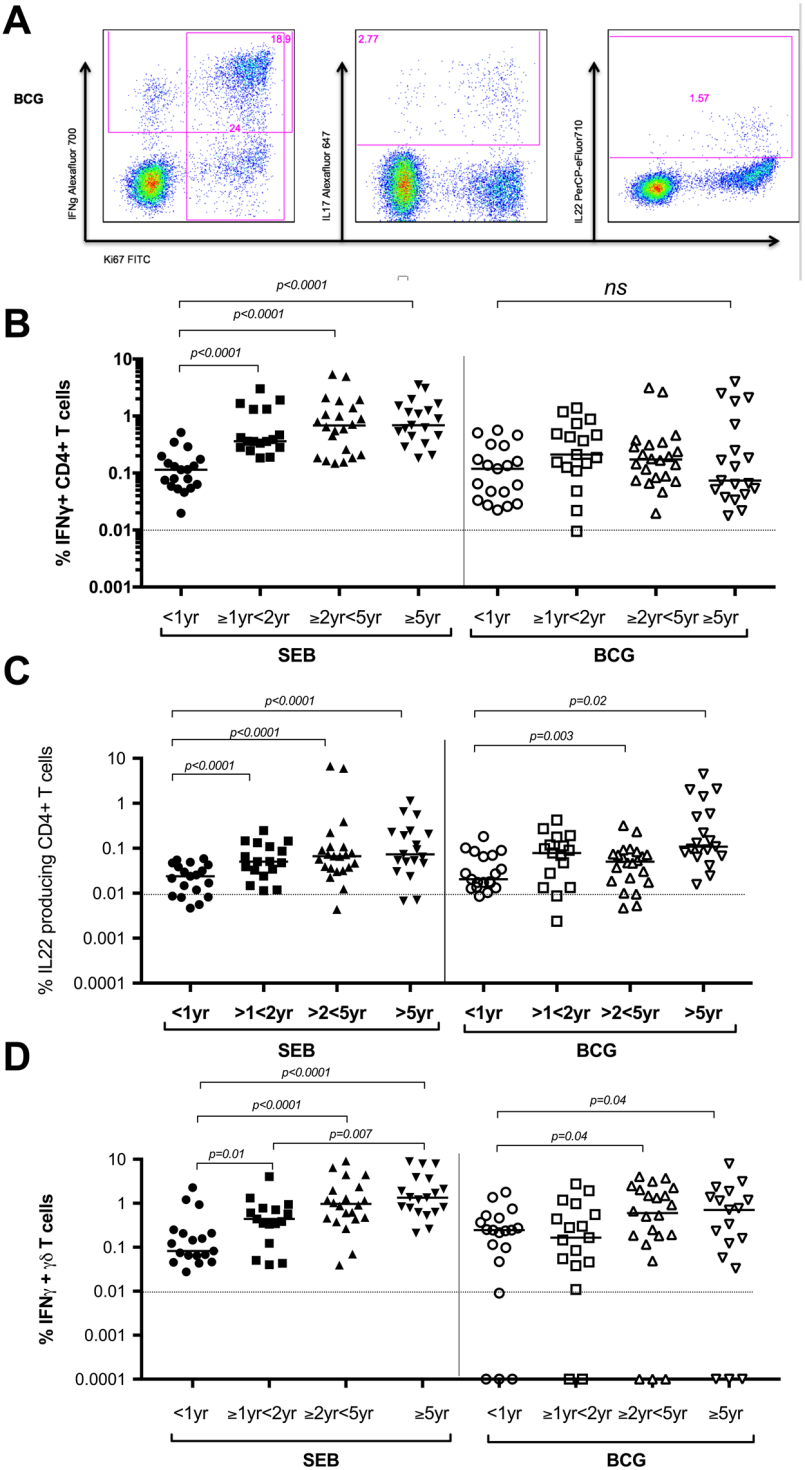
Children of all ages have measurable BCG specific CD4+ IFNγ responses, but non-specific responses increase with increasing age. (**A**) Flow cytometric plots from a representative individual infant showing intracellular IFNγ, IL17 or IL22 cytokine secretion by BCG stimulated CD4+ T cells are shown. (**B**) Frequencies of IFNγ producing CD4+ T cells, (**C**) IL22 producing CD4+ T cells or (**D**) IFNγ producing γδ T cells, as detected by an intracellular cytokine assay following stimulation of whole blood with SEB or BCG for 20 hours, are shown. Horizontal bars represent median values. The dotted line represents a cut-off of 0.01% for a significant response. The Mann-Whitney test was used to calculate p values.

To examine the age-dependent T cell effector responses to BCG or SEB stimulation *in vitro*, we measured the magnitude of responses by flow cytometry and quantified CD4+, CD8+ and γδ + T cells expressing IFNγ, IL17 and IL22. Following stimulation with BCG *in vitro*, BCG specific T cell responses were observed in all children, in particular all children demonstrated measurable IFNγ CD4+ T cell responses with no significant differences between the age groups (Fig. 1B). Interestingly, in response to the positive control antigen SEB, there was a significantly lower proportion of IFNγ producing CD4+ T cells in infants compared to those in all older age groups (Fig. 1B). The same pattern of response to SEB was observed in γδ (Fig. 1D) and CD8+ T cell responses (Data not shown). BCG specific IFNγ producing γδ T cells were significantly higher in children over five years compared to those under a year of age (Fig. 1D) (>5 yrs median 0.7% [IQR 0.05–1.8] vs <1 yr median 0.25 [IQR 0.04–0.46]; p = 0.04).

IL22 expressing BCG specific CD4+ T cells were produced in all ages, and their frequency were noted to be significantly higher in children over two years of age compared to infants (Fig. 1C). In contrast, BCG specific IL22 producing γδ T cell frequencies were higher in infants under 1 year of age compared to older infants (<1 yr median 0.245 [IQR 0.07–0.78] vs >5 yr median 0.05 [IQR 0.015–0.12]; p = 0.02) (data not shown). BCG specific IL17 production was low in all T cell types with a high number of non-responders (data not shown).

### BCG-induced secreted cytokine levels demonstrate decreased pro-inflammatory responses in infants

**Figure 2:**
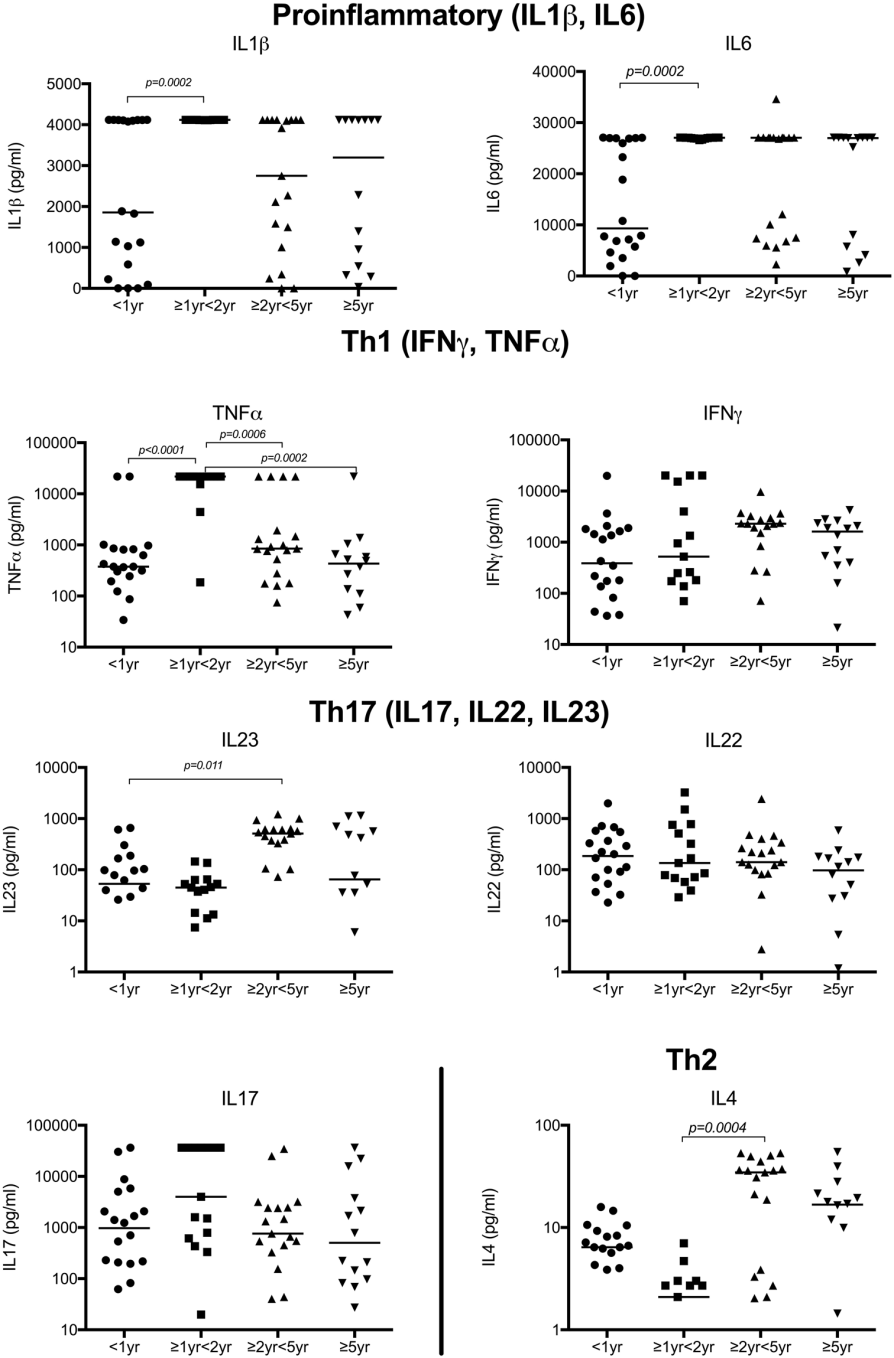
Secretion of IL1β, IL6 and TNFα was significantly lower in infants than older children. Cytokines were measured in supernatants from diluted whole blood cultures, stimulated with BCG for 6 days using a multiplex assay. Horizontal bars represent median values. Data shown on a log scale. A Mann Whitney test was used to calculate p values.

To explore functional properties of the T cells involved in Th1/Th17 pathways, we measured pro-inflammatory and effector cytokines secreted following BCG stimulation of whole blood *in vitro* for 6 days. The levels of IL1β, IL6, IL23 and TNFα were lower in children <1 yr of age compared to older children (Fig. 2), but there were no differences in levels of IFNγ, IL17 or IL22. There was a trend to increasing levels of IL4 with age, only reaching significance between children ≥1 < 2 yrs and those ≥2 < 5 yrs of age (Fig. 2). There were no significant differences in the other cytokines measured as part of this panel (IL10, IL21, IL31, IL33 or sCD40L; data not shown)

### Influence of age on proliferative capacity of T cells following BCG stimulation for 6 days

**Figure 3:**
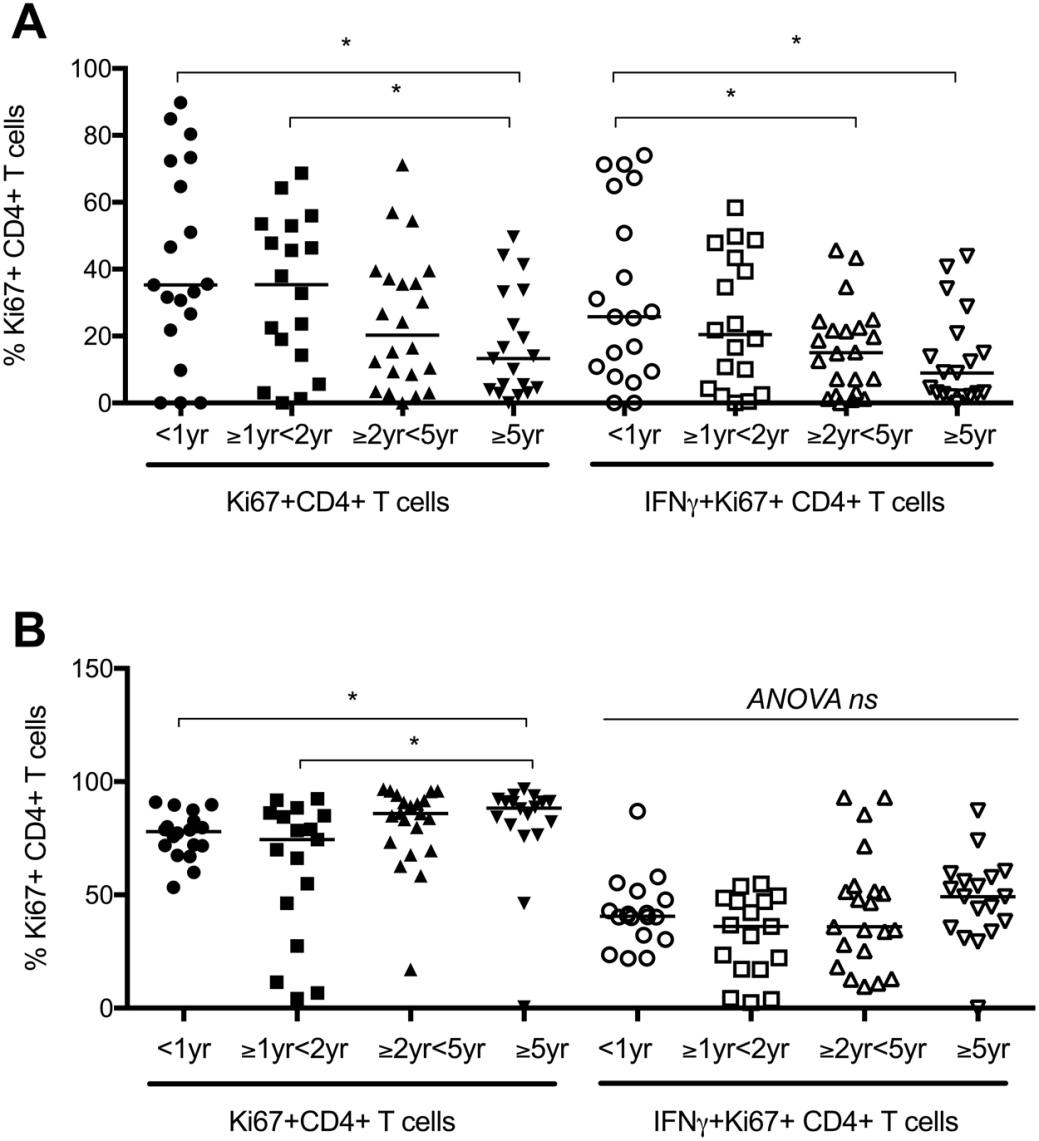
The frequencies of Ki67+ CD4 and IFNγ expressing Ki67 CD4+ T cells following stimulation with BCG (**A**) or SEB (**B**) for 6 days are shown. Ki67 proliferation and expression of IFNγ in response to BCG stimulation *in vitro* wanes with increasing age, however non-specific proliferation due to SEB increases with increasing age. Horizontal bars represent median values.

In order to determine potential differences in antigen-specific proliferative capacity and between the four age groups, a six day lymphoproliferation assay using Ki67 was carried out. The frequency of BCG stimulated proliferating CD4+ T cells decreased with increasing age, with significantly fewer cells in children >5years of age compared to infants and children under 2 years of age (Fig. 3A). To explore potential functional differences, key intracellular cytokines produced by BCG stimulated Ki67+ CD4+ T cells were also measured. Antigen-specific intracellular IFNγ decreased significantly with increasing age (Fig. 3A). The same pattern was noted in γδ and CD8+ T cells (data not shown). The median frequencies of IL17 and IL22 production by Ki67+ T cells of all phenotypes were low with a high number of non-responders, and there were no significant differences between the age groups (data not shown).

To determine whether these responses represented antigen-specific or generic responses, Ki67 proliferative responses, including cytokine expression, were also measured following stimulation with SEB. There was a significant increase in proliferative capacity of CD4+ T cells in response to SEB with increasing age, in contrast with the BCG specific responses which decreased with increasing age. There were no significant differences in intracellular cytokines produced by proliferating cells with increasing age (Fig. 3B).

No significant differences were seen in the proliferative response or cytokine production of γδ or CD8+ T cells in the four age groups following 6-day stimulation with SEB (Data not shown).

### Regulatory T cells in immune response to mycobacteria

**Figure 4:**
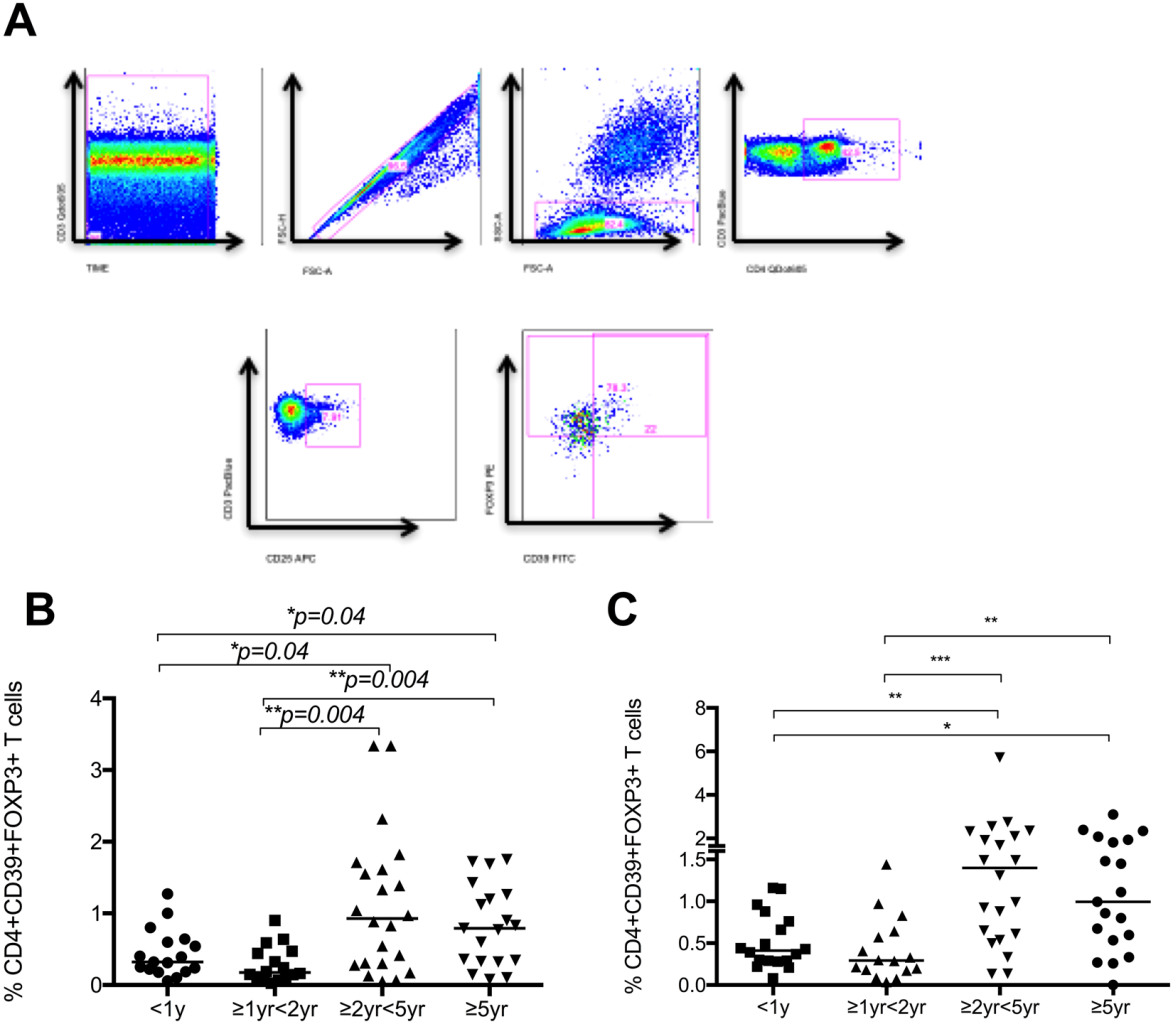
Regulatory T cells (**A**) Representative plots from a healthy control child are shown. From left to right, CD3 vs Time is shown to detect differences in flow. Cell doublets were excluded with forward scatter area versus forward scatter height parameters. Plotting FSC-A vs SSC-A allows identification of the lymphocyte population based on size and granularity. CD4+CD3+T cells were identified, followed by CD4+CD25+T cells. Using FMO gates, FOXP3 and CD39 gates were placed to identify the CD3+CD4+CD25+CD39+FOXP3+ population. Age-related frequency of regulatory T cells in (**B**) unstimulated whole blood and (**C**) whole blood incubated with BCG for 20 hours. Horizontal bars represent median values.

To determine the influence of age on circulating regulatory T cells in unstimulated and BCG stimulated whole blood, the frequency of CD4+CD25+CD39+FOXP3 T cells was measured in all four age groups. The frequency of regulatory T cells was found to increase significantly with increasing age (Fig. 4). To determine whether these regulatory T cells had a functional correlate, we measured the levels of IL10 in plasma collected from whole blood stimulated with BCG for 6 days and correlated regulatory T cells as well as Th1/Th17 cell phenotypes. No significant differences were observed. Furthermore, there were no significant correlations between regulatory T cells and frequency of Th1 or Th17 cytokine expression in CD4+, CD8+ or γδ T cells between any of the age groups. (Data not shown).

### Memory phenotype of BCG specific CD4 T cells

**Figure 5:**
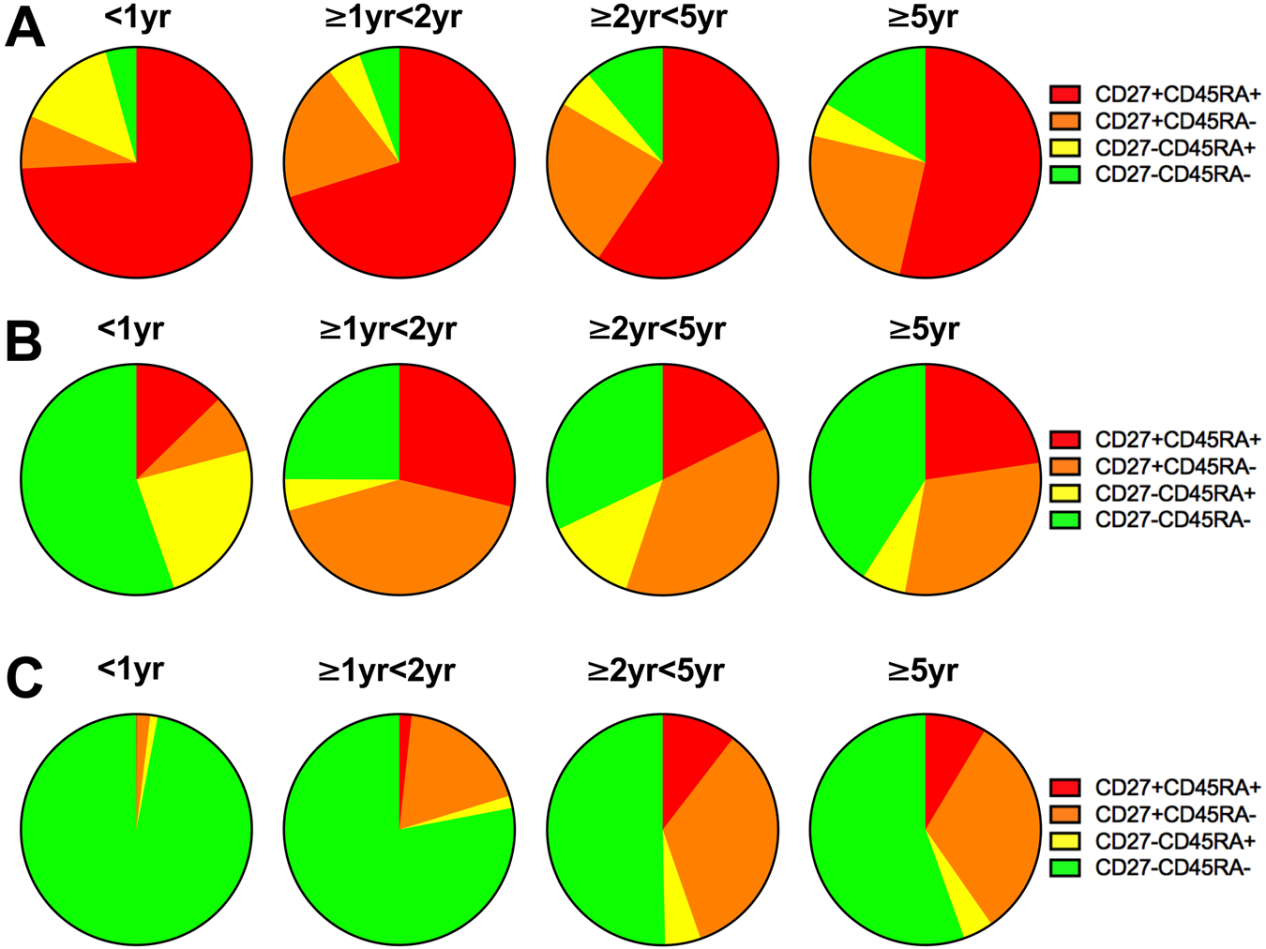
(**A**) The frequency of CD4+ T cell memory phenotypes as described by expression of CD27 and CD45RA in the four age groups is shown. (**B**) Memory phenotype of BCG specific IFNγ expressing CD4+ T cells in children of different age groups after 20 hours stimulation with BCG and (**C**) after 6 days stimulation with BCG.

Given the age-related differences in the proliferative capacity and cytokine production of T cells, in particular CD4 T cells, we next investigated whether these were associated with shifts in the CD4 T cell memory phenotype. The T cell memory phenotype in unstimulated whole blood was defined by expression of CD27 and CD45RA on the cell surface. Conventionally, dual positive cells (CD27+CD45RA+) are considered naïve; expression of CD27 alone (CD27+CD45RA−) is described as a central memory population; the absence of CD27 allows cells to migrate to the site of infection, those that express neither marker (CD27−CD45RA−) are described as effector memory T cells, while those that re-express CD45RA (CD27−CD45RA+) are terminally differentiated memory cells. A naïve CD4 T cell phenotype, CD27+CD45RA+, predominated in all age groups and decreased from 80% in infants to 49% in children older than 5 years (p < 0.0001) (Fig. 5A).

As expected, IFNγ was produced by antigen-specific CD4+ T cells. Although the patterns differed and there was a trend to fewer effector T cells with increasing age, there were no significant age-related differences between the four memory phenotypes (Fig. 5B).

The memory phenotype of proliferating CD4+ T cells was determined by measuring expression of CD27 and CD45RA surface markers. In infants, the majority of the cytokine producing Ki67 proliferating CD4+ T cells following BCG stimulation *in vitro* were expressing an effector memory phenotype (CD27−CD45RA−). In contrast, in older children, there was greater heterogeneity with increased representation of the naïve (CD27+ CD45RA+) and central memory (CD27+CD45RA−) phenotypes (Fig. 5C) (CD27−CD45RA− expressing Ki67+CD4 T cells – infants <1 yr median 96.8% (IQR 90.6–98.4) vs children >5 yr median 44.7% (IQR 30.3–68.2); p < 0.0001). The memory phenotypes of all CD4+ and γδ T cells producing cytokines (IFNγ, IL17 and IL22) were similar (data not shown).

## Discussion

This study sought to expand our understanding of phenotypic and functional characteristics of T cell populations in relation to BCG vaccination beyond infancy and including CD4, CD8, γδ and regulatory T cells and relevant cytokines in children. We found significant changes in antigen-specific and non-specific T cell responses related to age and time since receiving BCG vaccination with a contrasting pattern induced by antigen-specific and non-specific stimulation *in vitro*. Whilst waning efficacy of BCG is well described in the literature^15,16^, the underlying mechanisms have remained unclear. Our data, in South African children, support the notion that waning immunity to BCG over time could be due to waning of BCG-specific proliferation and expansion of effector CD4 T cells with age, associated with an increase in regulatory T cells.

Our data confirmed that healthy, BCG vaccinated children of all ages have measurable CD4+ T cell effector responses to BCG^17^. Conventional T cell effector responses, as defined by IFNγ producing CD4+ T cells, are an integral part of the immune response to mycobacteria. However recent studies demonstrate that IFNγ producing CD4+ T cells induced by either BCG or the TB vaccine candidate MVA85A, do not correlate with protective immunity^5,18,19^.

Based on results from animal studies, we anticipated that infants would have more robust Th17-associated responses than older children, measured as IL17 and IL22 production in response to BCG stimulation in our study. However, our data show that although IL17 was produced by both CD4+ and γδ T cells in response to BCG stimulation *in vitro* in some children, there were no clear patterns, with IL 17 levels either low or non-measurable in each age group. In contrast, the production of IL22 by γδ T cells was higher in younger children and decreased in older children in parallel with increasing IFNγ levels.

Despite the robust antigen specific CD4 T cell responses, other measures of immunity- such as lower non-specific SEB induced T cell responses, lower levels of BCG-induced IL1β, IL6 and TNFα and BCG-induced γδ T cell responses- were distinctly different in infants compared to older children. Previous studies of healthy BCG vaccinated infants have measured production of TNFα and IL6 as measures of non-adaptive responses following stimulation with TLR agonists or PPD in whole blood *in vitro*, but did not include children older than 12 months of age^20,21^. Shey *et al*. demonstrated increasing levels of TNFα, IL6 and IL12p40 by BCG stimulated monocytes from birth to 9 months, but did not explore these responses in older children^22^. In contrast, a further study of TLR responses in neonates and children up to 2 yrs of age in comparison to adults showed that IL6 and IL23 levels fell from birth to 2 yrs, by which time they were comparable to adult levels^23^. These differences may be explained by a shorter stimulation period (6 or 18 hours vs 6 days) and the use of specific TLR agonists rather than live BCG, which is likely to elicit different patterns of responses.

Limitations in the adaptive immune responses of infants are thought to reflect the naive state of most of their circulating T cells. Although the infants in this cohort had increased numbers of naïve T cells *ex-vivo*, these were able to differentiate into effector and central memory phenotypes in response to BCG stimulation. BCG specific CD4+ T cells from children of all ages in our cohort displayed phenotypic characteristics of both central memory and effector memory T cells, as previously shown by Soares *et al*. in children of up to 1 year of age^15^, possibly due to ongoing antigenic stimulation due to persistence of BCG, non-tuberculous mycobacteria(NTM) or indeed *Mycobacterium tuberculosis* in this highly endemic setting. However, when we examined the memory phenotype of BCG specific proliferating T cells, significant differences in the pattern of differentiation of these T cells between infants and older children were found. Following 6 days incubation of whole blood with BCG, almost the entire BCG specific T cell population in infants expressed an effector memory phenotype, whereas older children expressed a mixture of all memory phenotypes, with a greater central memory population.

The current literature has few and conflicting data regarding the relationship between regulatory T cells and age. We found that the frequency of circulating regulatory T cells increases with increasing age, both in unstimulated and BCG stimulated blood. Two South American studies have shown an inverse correlation between CD4+CD25+Foxp3+T cells and patient age in children from 1 yr to 19 yrs of age^24,25^. However, a Japanese group found that numbers stabilized and did not change after the first few days of life^26^. Recent work has shown significant differences in TLR innate immune responses in infants across four continents^27^, and it is likely that regional differences in regulatory T cell profiles also exist. Of note, IL10, a cytokine often produced by regulatory T cells, was noted to be decreased in the South African cohort compared to others in that study. One hypothesis to explain lower levels of regulatory T cells in the younger children in this cohort may be that BCG vaccination in the newborn period induces a strong Th1 response which is known to suppress regulatory T cell numbers. Regulatory T cells have previously been shown to be increased in adults and children with tuberculosis disease^8,28–30^ and are present in higher numbers in extra-pulmonary compared to pulmonary disease^8,31,32^, hence BCG induced suppression of regulatory T cells may indicate a mechanism of protection. Further studies including BCG unvaccinated cohorts would need to be conducted to conclude on this hypothesis. This study did not explore a direct functional effect of regulatory T cells on BCG induced CD4+ or γδ T cell responses, but it would be of interest to explore this *in vitro* or in an animal model, in particular focusing on the potential effect of regulatory T cell attenuation in the context of a booster dose of BCG vaccine^33^.

BCG vaccine appears to protect infants and young children only from disseminated disease in high endemic settings, and the short lived duration of protection has been attributed to either its failure to induce long lived memory cells or a gradual loss of BCG specific T cells^34^. Our data are in keeping with a gradual loss of the capacity to expand and differentiate BCG-specific effector T cells following re-stimulation with mycobacteria with increasing age. Boosting the BCG vaccine response, either through BCG revaccination or with a novel vaccine, may have a beneficial role in high endemic settings. There has however been controversy surrounding the role of BCG revaccination. Initial data from a large adolescent revaccination study did not report a protective benefit^35^. However data from longer term follow up show evidence of protection in some subpopulations^36,37^, in particular children under the age of 11, living in regions with lower NTM exposure. More recently, Dye *et al*. estimated the potential benefit of revaccinating adolescents with BCG and concluded that it could be both cost effective and efficacious in some settings^38^. Recently, in a prevention-of-infection Phase 2 trial of the novel vaccine candidate H4:IC31 conducted in South African adolescents, revaccination with BCG significantly reduced TB infections^39^. Revaccination is not associated with an increased risk of adverse events^40^, and there may be beneficial heterologous effects^41^.

Our study has some limitations: our data are cross-sectional rather than longitudinal and it would have been interesting to monitor changes in individuals over time. However, the associated frequent blood tests and the required time frame present an obstacle to such longitudinal work. In our cohort, BCG is likely to have ‘primed’ the immune response, resulting in robust antigen specific T cell responses, but we could not include an unvaccinated cohort, as this would be unethical in a highly endemic setting such as in South Africa.

In summary, our data confirm that infants mount comprehensive T cell responses but that recall responses to BCG wane with age. There are distinct differences in the innate immune responses and circulating regulatory T cell levels in infants compared to older children that may contribute to susceptibility to mycobacteria and warrant further investigation. As central memory responses to BCG are affected by age, revaccinating children before adolescence with its associated peak in TB disease might be beneficial in high endemic areas.
